# Association between the number of antibiotic types used and in-hospital mortality in patients with chronic kidney disease stage 5: A propensity score-matched study

**DOI:** 10.1097/MD.0000000000049833

**Published:** 2026-07-17

**Authors:** Qiantu Hu, Xuejiao Fan, Qiuhua Huang, Feixiang Lao, Yongxue Huang, Zhongqiu Mei, Qianqian Luo, Suzhen Wei, Qianni Huang, Limei Ou

**Affiliations:** a Department of Hospital Infection Management, Guangxi Zhuang Autonomous Region People’s Hospital, Nanning, China; b Department of Preventive Medicine, Guangxi Zhuang Autonomous Region People’s Hospital, Nanning, China; c Department of Nephrology, Guangxi Zhuangrong Region People’s Hospital, Nanning, China.

**Keywords:** antibiotic use, chronic kidney disease stage 5 (CKD5), in-hospital mortality, propensity score matching (PSM)

## Abstract

This study aimed to investigate the association between antibiotic use and in-hospital mortality among patients with chronic kidney disease stage 5 (CKD5), to inform optimized antibiotic stewardship. A retrospective study was conducted on patients diagnosed with CKD5 in the nephrology department of a tertiary hospital in Guangxi, China, from January 2022 to December 2024. Patients with in-hospital mortality were matched with surviving controls using propensity score matching. Firth penalized logistic regression was employed to reduce small-sample bias, and *E*-values were calculated to assess robustness to unmeasured confounding. Indicators of antibiotic use were then analyzed and compared between the 2 groups. A total of 3359 CKD5 patients were enrolled, with an in-hospital mortality rate of 0.98%. After propensity score matching (31 in-hospital mortality matched with 62 survivors), multivariable analysis showed that the number of antibiotic types used was significantly associated with in-hospital mortality (Firth odds ratio = 1.650, 95% CI: 1.148–2.492, *P* = .006; *E*-value = 1.889). This association was further supported by categorical analysis, where most antibiotic type categories showed significantly elevated mortality odds compared with no antibiotic use. Duration of antibiotic use (DOAU) was also significantly associated with mortality, though the effect size was modest (odds ratio = 1.081, 95% CI: 1.003–1.173, *P* = .041). Multivariate regression analysis further clarified the factors influencing antibiotic use indicators: types of antibiotics used was significantly associated with number of pathogenic species, total duration of fever, and number of venous catheterizations. Separately, DOAU was associated with total duration of fever, ICU days, and number of pathogenic species . The number of antibiotic types used is independently associated with in-hospital mortality in patients with CKD5, with DOAU showing a modest but significant association. These results support more cautious antibiotic stewardship in patients with CKD5, including improving etiological testing before antibiotic administration and avoiding unnecessary increases in the number of antibiotic types used. Nevertheless, these findings warrant validation in larger prospective cohorts.

## 1. Introduction

Chronic kidney disease (CKD) is a major contributor to global morbidity and mortality among noncommunicable diseases. Globally, the total number of CKD cases across all stages reached 697.5 million in 2017, with 1.2 million deaths attributed to this condition.^[1]^ Notably, the prevalence of CKD is also alarmingly high in China. A large nationwide representative cross-sectional study conducted between 2018 and 2019 estimated that the prevalence of CKD in mainland China was 8.2%, corresponding to approximately 82 million adult patients. Furthermore, the mortality rate associated with CKD has risen to 10.6%,^[2]^ making it a critical public health concern in the country.^[3]^ Additionally, studies have indicated a significant increase in the proportion of deaths caused by renal failure among patients with CKD5, soaring from 2–4% to 9–10%.^[4]^ This evidence highlights the need to investigate the underlying causes of renal failure and mortality-related risk factors in patients with CKD5, to inform the development of targeted preemptive interventions for mortality reduction.

Studies have demonstrated that antibiotic use is associated with increased renal burden and subsequent renal failure in CKD5 patients.^[5,6]^ Impaired glomerular filtration function in these patients leads to delayed antibiotic excretion; furthermore, the increased number of antibiotic classes used and polypharmacy with multiple antibiotics can further induce nephrotoxic effects,^[7]^ ultimately contributing to patient mortality. This highlights the need to investigate the association between antibiotic use and mortality in CKD5 patients.

CKD5 patients not only are at higher risk for severe acute kidney injury but also face a poorer prognosis once it occurs. Progression to end-stage renal disease imposes a substantial burden on individuals, their families, and society at large. Due to disease-related immunosuppression and reduced resistance to infections, severe infections represent a direct contributing factor to premature mortality in this patient population.^[8]^ Additionally, invasive procedures are frequently performed during clinical management, necessitating antibiotic use for infection prophylaxis when clinically indicated.^[9]^ Collectively, these factors render antibiotic administration unavoidable in the clinical care of CKD5 patients, whether for therapeutic or prophylactic purposes. Notably, CKD patients face a heightened risk of inappropriate antibiotic therapy related to renal function.^[10]^ A prior study reported that 64% of antibiotic prescriptions were inappropriate among patients with CKD stages 4 to 5.^[11,12]^ Investigating the risk relationship between antibiotic use and mortality in CKD5 patients may provide evidence-based guidance for clinical medication, thereby reducing mortality risk and improving the quality of life of these patients.

However, there is a paucity of research investigating the association between antibiotic use and in-hospital mortality in CKD5 patients. Notably, no clinical studies have confirmed whether such an association exists. Therefore, this study aimed to conduct statistical analysis of antibiotic-related indicators in CKD5 patients who died in-hospital, providing foundational data to guide antibiotic use.

## 2. Materials and methods

### 2.1. Study design and subjects

This was a retrospective case-control study. Study samples were collected from the People’s Hospital of Guangxi Zhuang Autonomous Region, a provincial-level tertiary first-class hospital located in Guangxi Zhuang Autonomous Region, China. Eligible participants were patients admitted to the Department of Nephrology between January 1, 2022, and December 31, 2024, who were diagnosed with CKD 5.

### 2.2. Patient selection

Inclusion criteria:

- Diagnosis of CKD 5.

CKD5 was defined as an estimated glomerular filtration rate (eGFR) < 15 mL/min/1.73m^2^, calculated using the CKD-EPI equation, persisting for more than 3 months.

- Aged ≥ 18 years at the time of admission.
- Length of hospital stay ≥ 24 hours.

Exclusion criteria:

- Aged < 18 years at the time of admission.
- Length of hospital stay < 24 hours.

### 2.3. Data collection and definitions

Data for this study were extracted from the hospital’s clinical records system, with a primary focus on parameters related to antimicrobial use. The collected data specifically included the following metrics:

Basic information: age, year, gender, ICU days, creatinine, dialysis modality, complications, and hospital stay.

Invasive procedures: admission surgeries, incision classification, mechanical ventilation duration (MVD), number of mechanical ventilation episodes (NOMV), venous catheter duration (VCD), number of venous catheterizations (NOVC).

Infection indicators: hospital infection, infection site, number of pathogenic species (NOPS), total duration of fever (TDF), and number of consecutive fever days (NCFD).

Antimicrobial application: preoperative prophylactic antibiotics (PPA), course duration of postoperative antibiotics (CDOPA), types of antibiotics used (TOAU), and duration of antibiotic use (DOAU).

Study data were extracted from the hospital’s clinical records system (HIS) using the Nosocomial Infection Surveillance system (integrated with our HIS since 2014), which provided complete infection-related data with no missing values. However, creatinine and dialysis modality could not be extracted automatically and were manually supplemented only for the post-PSM cohort, with 100% completeness.

Antibiotic application indicator definitions:

• PPA: binary indicator (yes/no) of whether antibiotics were administered within 0.5 to 1 hour before surgery to prevent surgical site infection.
• CDOPA: the number of days antibiotics were continued after surgery for prophylactic purposes to prevent surgical-related infections. For example, the number of days cefazolin was administered prophylactically following peritoneal dialysis catheter implantation to prevent potential peritonitis.
• TOAU: the number of distinct antibiotic classes used therapeutically during hospitalization. Each antibiotic class was counted only once per hospitalization. In cases of combination therapy, each concurrently used antibiotic class was counted separately.
• DOAU: the total number of days any antibiotic was administered therapeutically during hospitalization. Days on which multiple antibiotics were used concurrently were counted as a single day.

### 2.4. Statistical analysis

Statistical analyses and data visualization were performed using Stata 18.0 (StataCorp LLC) and R 4.5.0 (TUNA Team, Tsinghua University, China) with the MatchIt package (v4.5.0). Continuous variables were presented as mean ± standard deviation (mean ± SD) if normally distributed, or as median with interquartile range (IQR) if non-normally distributed. Categorical variables were described as numbers and percentages (n, %). Intergroup comparisons were conducted using appropriate statistical tests. For continuous variables, the independent samples *t*-test was applied for normally distributed data, while the Mann–Whitney *U* test was used for non-normally distributed data. Comparisons of categorical variables were performed using the chi-square test, as appropriate.

Propensity score matching (PSM) was performed at a 1:2 ratio to balance baseline characteristics between patients who died in-hospital and those who survived. Matching covariates included age, gender, admission year, and number of admission surgeries. PSM was implemented via the matchit() function in the R MatchIt package, using the nearest-neighbor matching algorithm with a fixed 1:2 matching ratio and a caliper width of 0.2 times the standard deviation of the propensity score to enhance matching quality.

To assess the association between antibiotic use indicators and in-hospital mortality in the matched cohort, Firth penalized logistic regression was employed to reduce small-sample bias and address separation issues arising from the low number of outcome events (31 deaths after matching). The events per variable was calculated to evaluate model stability. Results were reported as odds ratios (OR) with 95% profile likelihood confidence intervals (CIs). To provide nonparametric uncertainty estimates, bootstrap resampling (1000 replications) was performed, with bootstrap 95% CIs calculated using the percentile method. *E*-values were computed for statistically significant associations to assess robustness to unmeasured confounding; the *E*-value represents the minimum strength of association that an unmeasured confounder would need to have with both the exposure and the outcome to explain away the observed association.

For the analysis of factors associated with antibiotic use indicators, multivariate linear regression was used for continuous outcomes (TOAU, DOAU, CDOPA), and multivariate logistic regression was used for the binary outcome (PPA). All models were adjusted for the following prespecified candidate predictors: creatinine, total duration of fever (TDF), ICU days, number of pathogenic species (NOPS), number of mechanical ventilation events (NOMV), and number of venous catheterizations (NOVC). For the linear regression models (TOAU, DOAU, and CDOPA as outcomes), Q–Q plots of residuals showed that points approximately followed the diagonal line; thus, linear regression was considered appropriate.

### 2.5. Ethics approval and patient consent

**Figure 1.**
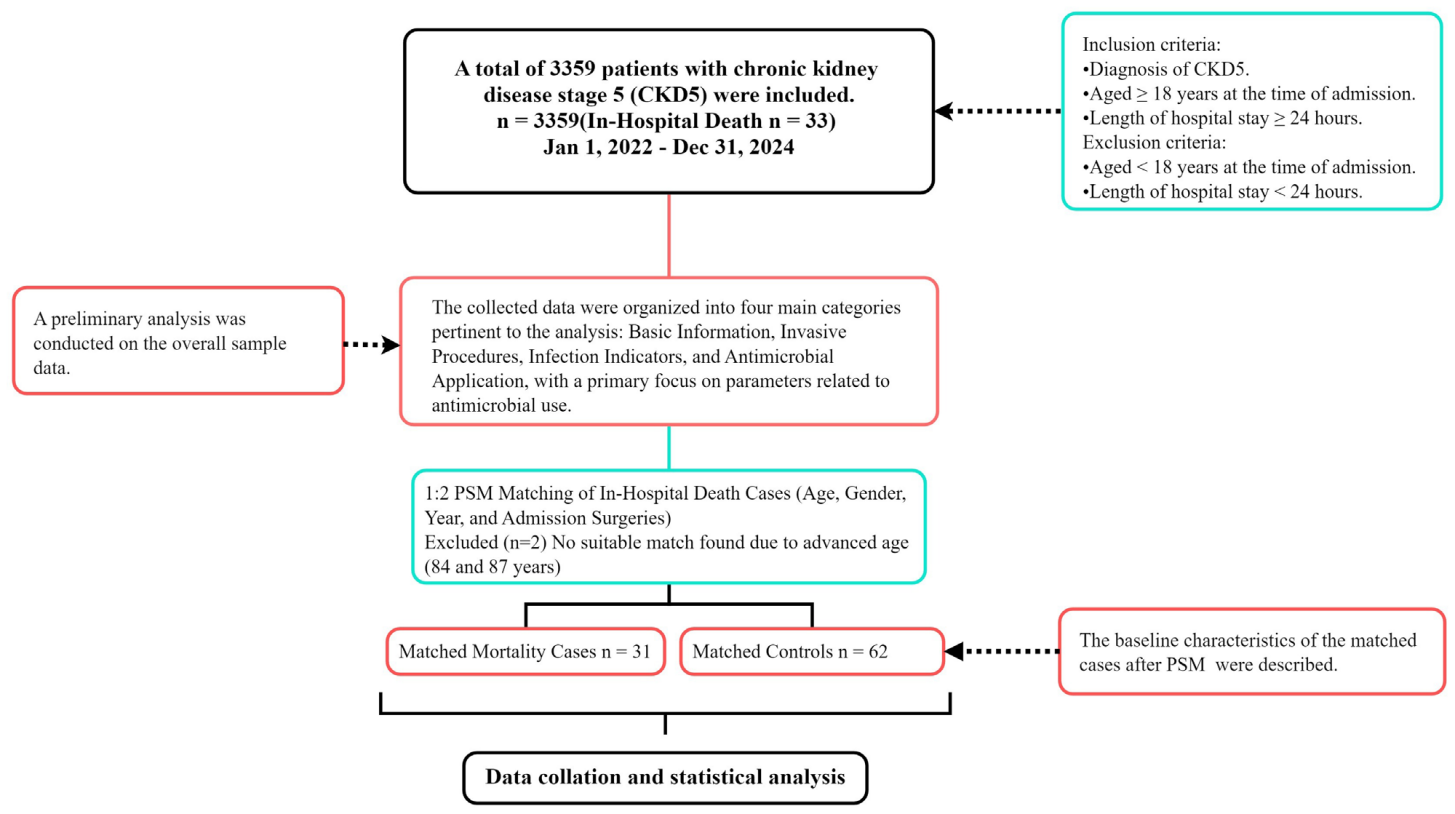
Flow diagram of patient selection, matching, and analysis. This diagram illustrates the retrospective identification and matching process for patients with chronic kidney disease stage 5 (CKD5). From an initial pool of 3359 eligible patients admitted between January 1, 2022, and December 31, 2024, 33 in-hospital mortality cases were identified. A 1:2 propensity score matching (PSM) was performed against 3326 survival controls, using age, gender, admission year, and admission surgeries as covariates. Two mortality cases (aged 84 and 87 years) were excluded from matching as no suitable controls could be found at the specified ratio. The final matched cohort consisted of 93 patients (31 mortality cases and 62 controls) for all subsequent statistical analyses. CKD5 = chronic kidney disease stage 5, PSM = propensity score matching.

This retrospective study was approved by the Science and Technology Ethics Committee of the Guangxi Zhuang Autonomous Region People’s Hospital (Approval No.: KY-IIT-2025-219). The committee waived the requirement for informed consent as the research involved the analysis of fully anonymized data. All procedures were conducted in accordance with the ethical standards of the Declaration of Helsinki and the approved protocol. The process of patient enrollment, matching, and exclusion is illustrated in Figure 1.

## 3. Results

### 3.1. Characteristics of the study population

A total of 3359 eligible patients with CKD5 were enrolled in this study, among whom 33 patients (0.98%) died in-hospital, and 3326 patients were discharged after remission following treatment. The distribution of various clinical indicators and the results of univariate logistic regression are shown in Table 1.

**Table 1:** Baseline characteristics of chronic kidney disease stage 5 patients.

Variables	Survival (n = 3326)	In-hospital death (n = 33)	OR (95% CI)	*P* value
Demographics				
Age (yr)	56.57 ± 14.04	70.79 ± 12.43	1.09 (1.06, 1.12)	**<.001**
Year (%)			0.86 (0.55, 1.32)	.481
2022	1041 (31.30)	10 (30.30)		
2023	1220 (36.68)	16 (48.48)		
2024	1065 (32.02)	7 (21.21)		
Gender (male, %)	1939 (58.3)	21 (63.64)	1.25 (0.61, 2.55)	.537
Hospital stay (d)	7.35 ± 8.08	17.33 ± 12.35	1.05 (1.03, 1.07)	**<.001**
ICU days (d)	0.08 ± 1.20	3.61 ± 6.21	1.24 (1.14, 1.34)	**<.001**
Complications (%, Diabetes/Hypertension/Anemia)			7.73 (3.28, 18.18)	**<.001**
No	3214 (96.63)	26 (78.79)		
Yes	112 (3.37)	7 (21.21)		
Invasive procedures				
Admission surgeries (n)	0.97 ± 0.58	0.48 ± 0.57	0.17 (0.08, 0.33)	**<.001**
Incision classification (%)			0.2 (0.1, 0.38)	**<.001**
Clean Incision	556 (16.72)	18 (54.55)		
Clean-contaminated Incision	2378 (71.50)	14 (42.42)		
Contaminated incision	392 (11.79)	1 (3.03)		
MVD (d)	0.04 ± 0.85	3.15 ± 5.38	1.37 (1.22, 1.55)	**<.001**
NOMV (n)	0.01 ± 0.19	0.73 ± 0.84	7.01 (4.44, 11.05)	**<.001**
VCD (d)	0.86 ± 10.86	2.61 ± 5.55	1.004 (0.99, 1.01)	.457
NOVC (n)	0.22 ± 0.68	1.45 ± 1.35	1.78 (1.47, 2.16)	**<.001**
Infection indicators				
Hospital infection (%)			1.54 (0.23, 10.49)	.659
No	3264 (98.14)	32 (96.97)		
One	60 (1.8)	1 (3.03)		
Two	2 (0.06)	0 (0)		
Infection site (%)			1.76 (1.42, 2.17)	**<.001**
NO	3094 (93.02)	22 (66.67)		
Hematologic system	83 (2.50)	1 (3.03)		
Respiratory system	46 (1.38)	5 (15.15)		
Urinary system	49 (1.47)	2 (6.06)		
Digestive system	37 (1.11)	1 (3.03)		
Skin/soft tissue	17 (0.51)	2 (6.06)		
NOPS (n)	0.08 ± 0.30	0.42 ± 0.66	3.71 (2.27, 6.08)	**<.001**
TDF (d)	0.23 ± 1.01	1.18 ± 2.13	1.32 (1.17, 1.5)	**<.001**
NCFD (d)	0.18 ± 0.74	1.00 ± 1.84	1.47 (1.24, 1.74)	**<.001**
Antibiotic application				
PPA (yes)	598 (17.98)	12 (36.36)	2.61 (1.28, 5.33)	**.009**
CDOPA (d)	1.08 ± 3.51	3.12 ± 7.59	1.06 (1.02, 1.11)	**.004**
TOAU (n)	0.58 ± 1.17	2.85 ± 2.11	1.76 (1.54, 2.01)	**<.001**
TOAU* (%)			2.20 (1.83, 2.64)	**<.001**
NO	2365 (71.11)	3 (9.09)		
1	452 (13.59)	8 (24.24)		
2	268 (8.06)	6 (18.18)		
3	124 (3.73)	5 (15.15)		
4	68 (2.04)	4 (12.12)		
≥5	49 (1.47)	7 (21.21)		
DOAU (d)	3.11 ± 6.85	12.45 ± 12.52	1.07 (1.05, 1.09)	**<.001**

Bold values indicate statistical significance (*P* < .05).

CDOPA = course duration of postoperative antibiotics, DOAU = duration of antibiotic use, MVD = mechanical ventilation duration, NCFD = number of consecutive fever days, NOMV = number of mechanical ventilation, NOPS = number of pathogenic species, NOVC = number of venous catheterizations, PPA = preoperative prophylactic antibiotics, TDF = total duration of fever, TOAU = types of antibiotics used, VCD = venous catheter duration.

* The same variable categorized. All data were showed as mean ± standard deviation or number of participants [percentage] appropriately. Univariate logistic regression was used to determine the association between various clinical indicators and in-hospital mortality among patients.

In patients with CKD5, the mean age of the in-hospital death group (70.79 ± 12.43 years) was higher than that of the survival group (56.57 ± 14.04 years). The average hospital stay of the in-hospital death group (17.33 ± 12.35 days) was significantly longer than that of the survival group (7.35 ± 8.08 days). The in-hospital death group also had a markedly longer ICU stay (3.61 ± 6.21 days) compared to the survival group (0.08 ± 1.20 days). Regarding complications, the proportion of patients with diabetes, hypertension, or anemia was significantly higher in the in-hospital death group (21.21%) than in the survival group (3.37%). Regarding invasive procedures, the in-hospital death group had a lower Admission Surgeries rate (see Table S1, Supplemental Digital Content 1, which presents the aggregated surgical procedures and surgical treatment regimens in the overall cohort, stratified by survival and in-hospital death groups), and incision classification was associated with in-hospital outcomes, Furthermore, the the mortality group had higher rates of MVD, NOMV, and NOVC compared to the survival group; among infection-related indicators, infection site distribution, NOPS, fever-related indicators (TDF, NCFD) showed associations with in-hospital outcomes; in terms of antibiotic application (see Table S2, Supplemental Digital Content 2, which details antibiotic utilization in each group in the overall cohort), the use of PPA, the CDOPA, the TOAU, and the DOAU in the mortality group were all different from those in the survival group. When TOAU was categorized, a progressive increase in mortality risk was observed with an increasing number of antibiotic types used. Indicators with no statistical differences between the 2 groups included year, gender, VCD, and hospital-acquired infection.

The results of univariate Logistic regression analysis showed that age (OR = 1.09, *P* < .001), hospital stay (OR = 1.05, *P* < .001), ICU days (OR = 1.24, *P* < .001), presence of complications (OR = 7.73, *P* < .001), admission surgeries (OR = 0.17, *P* < .001), incision classification (OR = 0.20, *P* < .001), MVD (OR = 1.37, *P* < .001), NOMV (OR = 7.01, *P* < .001), NOVC (OR = 1.78, *P* < .001), infection site (OR = 1.76, *P* < .001), NOPS (OR = 3.71, *P* *<* .001), TDF (OR = 1.32, *P* < .001), NCFD (OR = 1.47, *P* < .001), PPA use (OR = 2.61, *P* = .009), CDOPA (OR = 1.06, *P* = .004), TOAU (OR = 1.76, *P* < .001), categorized TOAU (OR = 2.20, *P* < .001), and DOAU (OR = 1.07, *P* < .001) were all significantly associated with in-hospital mortality. However, year of hospitalization (OR = 0.86, *P* = .481), gender (OR = 1.25, *P* = .537), VCD (OR = 1.004, *P* = .457), and hospital-acquired infection (OR = 1.54, *P* = .659) had no significant association with mortality.

### 3.2. Propensity score matching and comparative analysis after matching

A 1: PSM method was employed to match control subjects with patients who experienced in-hospital mortality, with matching variables including age, gender, year, and admission surgeries. Initially, 33 in-hospital mortality cases were matched to 64 control cases. However, 2 mortality cases (aged 84 and 87 years) were excluded as no suitable controls could be matched at a 1:2 ratio using the defined criteria, a limitation attributable to their advanced age. Ultimately, 31 mortality cases and 62 control cases were included in the subsequent analyses.

**Figure 2.**
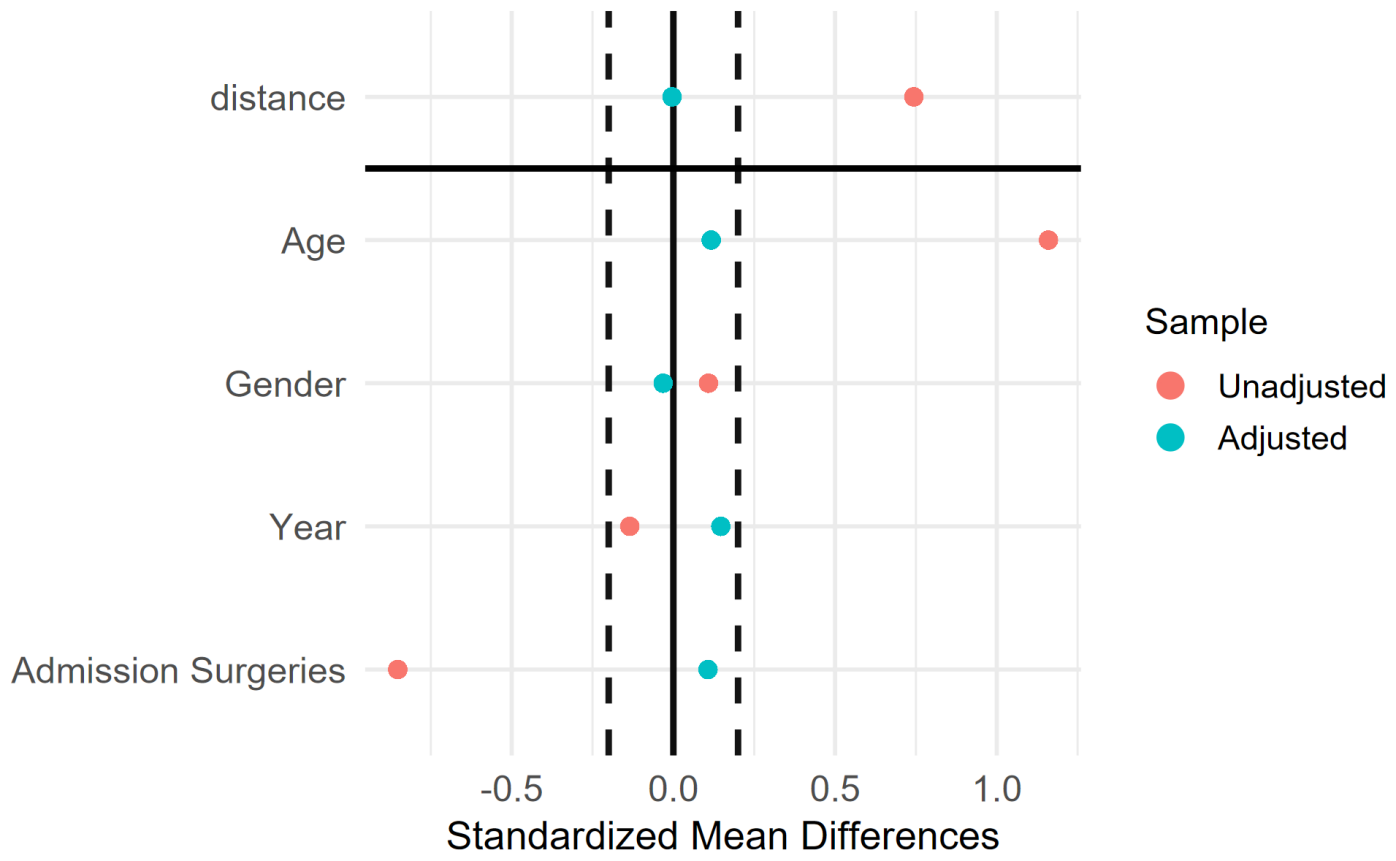
Covariate balance before and after propensity score matching (PSM). Standardized mean differences (SMDs) for selected covariates (age, gender, year, and admission surgeries) are plotted before (red circles) and after (teal circles) PSM. The vertical dashed lines indicate the commonly accepted balance threshold of |SMD| < 0.2. After matching, all covariates achieved adequate balance (|SMD| <0.2), indicating successful reduction of observed confounding. PSM = propensity score matching, SMD = standardized mean difference.

To assess covariate balance, standardized mean differences (SMDs) were calculated before and after PSM (Fig. 2). Prior to matching, substantial imbalances (|SMD| > 0.5) were observed for age (SMD > 1.0) and admission surgeries (SMD < −0.5), with milder discrepancies noted for gender and year. Following PSM, all measured covariates achieved adequate balance, with SMDs falling below the accepted threshold of 0.2. This indicates that PSM effectively reduced observed confounding, thereby strengthening the internal validity of subsequent comparative analyses.

After PSM (based on age, gender, year, and admission surgeries), 31 in-hospital deaths were compared with 62 matched survivors (see Table S3, Supplemental Digital Content 3, which presents the disaggregated surgical procedures and surgical treatment regimens in the propensity score-matched cohort, stratified by survival and in-hospital death groups). No significant differences remained in these matched baseline characteristics between the 2 groups (all *P* > .05). However, the in-hospital death group had a significantly longer hospital stay (median 13 vs 5.5 days, *P* < .001) and markedly longer ICU stay (3.58 ± 6.33 vs 0.05 ± 0.38 days, *P* < .001) compared with the survival group. No significant difference was observed in creatinine levels between the 2 groups (685.15 ± 300.30 vs 739.42 ± 289.38 μmol/L, *P* = .402); however, dialysis modality distribution differed significantly (*P* < .001), with hemodialysis being the predominant modality in both groups. The proportion of patients with complications (diabetes, hypertension, or anemia) was significantly higher in the in-hospital death group (22.58% vs 4.84%, *P* = .009). Indicators of invasive device utilization – including both duration (MVD and VCD) and frequency (NOMV and NOVC) – were all significantly higher in the mortality group (all *P* < .01). Furthermore, the mortality group exhibited more pronounced markers of infection: a higher NOPS, longer TDF, and NCFD (all *P* < .01). Infection site distribution showed borderline significance between groups (*P* = .067), with respiratory infections being more frequent in the in-hospital death group (16.13% vs 4.84%). Regarding antibiotic application (see Table S4, Supplemental Digital Content 4, which details antibiotic utilization in each group in the propensity score-matched cohort), the mortality group had a higher rate of PPA use, a longer CDOPA, a greater TOAU, and a longer DOAU (all *P* < .01). When TOAU was categorized, a progressive increase in mortality was observed with an increasing number of antibiotic types used (*P* < .001 for trend). Detailed results are presented in Table 2.

**Table 2:** Distribution of various indicators after propensity score matching.

Variables	Survival (n = 62)	In-hospital death (n = 31)	*P* value
Demographics			
Age (yr)	72 (62–77)	72 (62–78)	.806
Year (%)			.557
2022	26 (41.94)	10 (32.26)	
2023	23 (37.10)	15 (48.39)	
2024	13 (20.97)	6 (19.35)	
Gender (male, %)	39 (62.90)	19 (61.29)	.880
Hospital stay (d)	5.5 (4–9)	13 (8–21)	**<.001**
ICU days (d)	0.05 ± 0.38	3.58 ± 6.33	**<.001**
Creatinine (μmol/L)	739.42 ± 289.38	685.15 ± 300.30	.402
Dialysis modality (%)			**<.001**
Hemodialysis	60 (96.77)	30 (96.77)	
Peritoneal dialysis	2 (3.23)	1 (3.23)	
Complications (%, Diabetes/Hypertension/Anemia)			**.009**
No	59 (95.16)	24 (77.42)	
Yes	3 (4.84)	7 (22.58)	
Invasive procedures			
Admission surgeries (n)	0.45 ± 0.50	0.52 ± 0.57	.578
Incision classification (%)			.148
Clean incision	7 (25.00)	7 (46.67)	
Clean-contaminated incision	21 (75.00)	8 (53.33)	
MVD (d)	0 (0–0)	0 (0–6)	**<.001**
NOMV (n)	0.03 ± 0.25	0.77 ± 0.84	**<.001**
VCD (d)	0 (0–0)	0 (0–5)	**.002**
NOVC (n)	0 (0–0)	1 (0–2)	**<.001**
Infection indicators			
Hospital infection (%)			.613
None	61 (98.39)	30 (96.77)	
One	1 (1.61)	1 (3.23)	
Infection site (%)			.067
NO	55 (88.71)	20 (64.52)	
Hematologic system	1 (1.61)	1 (3.23)	
Respiratory system	3 (4.84)	5 (16.13)	
Urinary system	0 (0.00)	2 (6.45)	
Digestive system	2 (3.23)	1 (3.23)	
Skin/soft tissue	1 (1.61)	2 (6.45)	
NOPS (n)	0.13 ± 0.38	0.45 ± 0.68	**.004**
TDF (d)	0.16 ± 0.49	1.26 ± 2.18	**<.001**
NCFD (d)	0.16 ± 0.49	1.06 ± 1.88	**<.001**
Antibiotic Application			
PPA (yes)	9 (14.52)	12 (38.71)	**.009**
CDOPA (d)	0 (0–0)	0 (0–3)	**.007**
TOAU (n)	0.71 ± 1.19	2.74 ± 2.03	**<.001**
TOAU* (%)			**<.001**
NO	39 (62.90)	3 (9.68)	
1	11 (17.74)	8 (25.81)	
2	7 (11.29)	5 (16.13)	
3	3 (4.84)	5 (16.13)	
4	1 (1.61)	4 (12.90)	
≥5	1 (1.61)	6 (19.35)	
DOAU (d)	3.52 ± 5.94	11.39 ± 10.30	**<.001**

Bold values indicate statistical significance (*P* < .05).

CDOPA = course duration of postoperative antibiotics, DOAU = duration of antibiotic use, IQR = interquartile range, MVD = mechanical ventilation duration, NCFD = number of consecutive fever days, NOMV = number of mechanical ventilation, NOPS = number of pathogenic species, NOVC = number of venous catheterizations, PPA = preoperative prophylactic antibiotics, SD = standard deviation, TDF = total duration of fever, TOAU = types of antibiotics used, VCD = venous catheter duration.

* The same variable categorized. Data are presented as mean ± SD, median (IQR), or n (%). Group comparisons were made using *t*-tests, Mann–Whitney U tests, or χ^2^ tests, as appropriate.

### 3.3. Association between antibiotic-related indicators and in-hospital mortality

To investigate the correlation between antibiotic-related indicators and in-hospital mortality, Firth penalized logistic regression was employed to reduce small-sample bias and address separation issues (events per variable = 3.88). All models were adjusted for prespecified covariates: creatinine, complications, ICU days, NOPS, NOMV, NOVC, and dialysis modality. Due to high collinearity between NOPS and infection site (correlation coefficient = 0.860, *P* < .001), which prevented model convergence when both were included simultaneously, only NOPS was retained as a covariate to represent infection severity.

When TOAU was analyzed as a continuous variable, it remained significantly associated with in-hospital mortality after multivariable adjustment (Firth OR = 1.650, 95% CI: 1.148–2.492, *P* = .006; bootstrap 95% CI: 1.143–3.915).

When TOAU was categorized (with no antibiotic use as the reference), a generally increasing trend in mortality risk was observed with higher numbers of antibiotic types, although the association was not strictly monotonic. Compared with patients who received no antibiotics, those who used 1 type (OR = 5.653, 95% CI: 1.184–31.496, *P* = .030), 2 types (OR = 7.489, 95% CI: 1.317–47.817, *P* = .024), 4 types (OR = 21.082, 95% CI: 1.985–347.478, *P* = .012), or 5 or more types (OR = 14.303, 95% CI: 1.445–185.273, *P* = .023) had significantly higher odds of in-hospital mortality. The association for 3 types did not reach statistical significance (OR = 3.845, 95% CI: 0.185–40.395, *P* = .327). Bootstrap CIs were consistent with these findings, albeit wider due to the small-sample size.

Regarding other antibiotic indicators, DOAU was also significantly associated with in-hospital mortality in the multivariable Firth model (OR = 1.081, 95% CI: 1.003–1.173, *P* = .041; bootstrap 95% CI: 1.012–1.206). In contrast, PPA (OR = 1.526, 95% CI: 0.396–5.432, *P* = .524) and CDOPA (OR = 0.940, 95% CI: 0.797–1.236, *P* = .452) were not significantly associated with in-hospital mortality after multivariable adjustment.

Sensitivity analyses using standard logistic regression yielded consistent results, though with slightly larger effect estimates: TOAU (OR = 1.857, 95% CI: 1.237–2.961) and DOAU (OR = 1.092, 95% CI: 1.009–1.192). *E*-values were calculated to assess robustness to unmeasured confounding. For TOAU, the *E*-value for the point estimate was 1.889, indicating that an unmeasured confounder would need to be associated with both TOAU and mortality by a risk ratio of 1.89-fold each to explain away the observed association; the *E*-value for the lower confidence limit was 1.348. For the significant TOAU categories, *E*-values ranged from 4.187 to 8.652, suggesting moderate to high robustness to unmeasured confounding. For DOAU, the *E*-value was 1.243 (lower confidence limit *E*-value = 1.041), indicating relatively lower robustness compared with TOAU. Detailed results are presented in Table 3.

**Table 3:** Association between antibiotic-related indicators and in-hospital mortality in CKD stage 5 patients after propensity score matching: results from firth logistic regression.

	OR (95% CI)	*P* value	Bootstrap (95% CI)	Logistic OR (95% CI)	*E*-value (estimate/LL)
TOAU	1.650 (1.148–2.492)	**.006**	(1.143–3.915)	1.857 (1.237–2.961)	1.889/1.348
TOAU*					
1	5.653 (1.184–31.496)	**.030**	(1.008–97.114)	6.409 (1.101–46.139)	4.187/1.398
2	7.489 (1.317–47.817)	**.024**	(1.212–128.227)	8.910 (1.309–72.228)	4.917/1.559
3	3.845 (0.185–40.395)	.327	(0.003–147.960)	2.667 (0.054–51.036)	–
4	21.082 (1.985–347.478)	**.012**	(2.208–2561.657)	38.614 (2.401–1293.265)	8.652/2.168
≥5	14.303 (1.445–185.273)	**.023**	(0.279–1515.968)	30.472 (1.967–988.609)	7.025/1.695
DOAU	1.081 (1.003–1.173)	**.041**	(1.012–1.206)	1.092 (1.009–1.192)	1.243/1.041
PPA	1.526 (0.396–5.432)	.524	(0.239–8.144)	1.540 (0.354–6.084)	–
CDOPA	0.940 (0.797–1.236)	.452	(0.686–1.523)	0.994 (0.780–1.329)	–

Firth penalized logistic regression was used to reduce small-sample bias and address separation (events per variable [EPV] = 3.88). All models were adjusted for prespecified covariates: creatinine, complications, ICU days, NOPS (merged as 0 vs 1–2), NOMV, NOVC, and dialysis modality.

Odds ratios (ORs) and 95% confidence intervals (CIs) from Firth regression (profile likelihood) and standard logistic regression are shown. Bootstrap 95% CIs were obtained from 1000 resamples of the entire model-building process to provide nonparametric uncertainty estimates.

*E*-values are reported for statistically significant exposures (*P* < .05). The *E*-value represents the minimum strength of association (on the risk ratio scale) that an unmeasured confounder would need to have with both the exposure and the outcome to explain away the observed OR (point estimate) or to shift its CI to include 1 (CI-based). Larger *E*-values indicate greater robustness to unmeasured confounding.

Bold values indicate statistical significance (*P* < .05).

CI = confidence interval, CDOPA = course duration of postoperative antibiotics, DOAU = duration of antibiotic use, EPV = events per variable, ICU = intensive care unit, NOMV = number of mechanical ventilation, NOPS = number of pathogenic species, NOVC = number of venous catheterizations, OR = Odds ratio, PPA = preoperative prophylactic antibiotics, TOAU = types of antibiotics used.

* The same variable categorized, with 0 as the reference group.

### 3.4. Factors associated with antibiotic use indicators by multivariate regression

**Figure 3.**
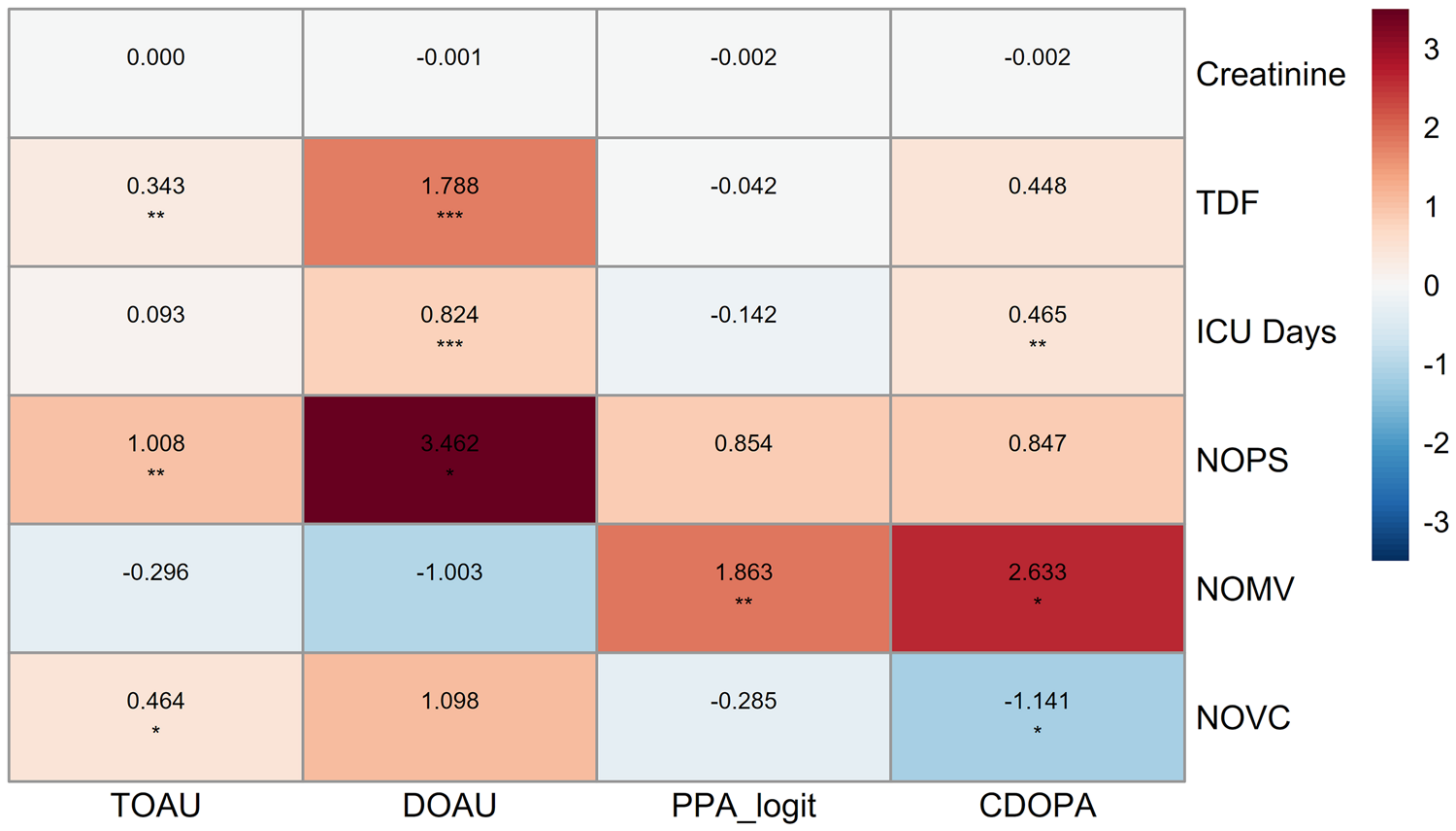
Heatmap visualizing multivariate regression coefficients of factors influencing antibiotic use indicators. Heatmap showing correlation coefficients from multivariate regression analyses for 4 antibiotic use indicators: multivariate linear regression was used for continuous outcomes (TOAU, DOAU, CDOPA), and multivariate logistic regression was used for the binary outcome (PPA_logit). All models were adjusted for the 6 candidate predictors listed in the table: creatinine, total duration of fever (TDF), ICU days, number of pathogenic species (NOPS), number of mechanical ventilation events (NOMV), and number of venous catheterizations (NOVC). Regression coefficients (Coef) represent the change in the outcome variable per unit increase in the predictor; for PPA_logit, coefficients are on the log-odds scale. Coefficients are color-coded (blue, negative association; red, positive association). Numerical values are the coefficients; significance markers denote *P < .05, **P < .01, ***P < .001. ICU = intensive care unit, NOMV = number of mechanical ventilation, NOPS = number of pathogenic species, NOVC = number of venous catheterizations, TDF = total duration of fever,

Multivariate regression analyses were conducted to identify factors associated with antibiotic use indicators in the propensity score-matched cohort. For the binary outcome PPA, logistic regression was used; for continuous outcomes (TOAU, DOAU, CDOPA), linear regression was applied. All models included 6 candidate predictors: creatinine, TDF, ICU days, NOPS, NOMV, NOVC. Detailed results, including regression coefficients and statistical significance, are presented as a heatmap in Figure 3 (see Table S5, Supplemental Digital Content 5, which illustrates the multivariable regression analysis of factors associated with various antibiotic use indicators).

The analysis revealed that TDF (coef = 0.343, *P* = .003), NOPS (coef = 1.008, *P* = .004), and NOVC (coef = 0.464, *P* = .021) were significantly and positively associated with TOAU. For DOAU, significant positive associations were observed for TDF (coef = 1.788, *P* < .001), ICU days (coef = 0.824, *P* < .001), and NOPS (coef = 3.462, *P* = .031). Regarding PPA, only NOMV showed a significant positive association (coef = 1.863, *P* = .007). For CDOPA, ICU days (coef = 0.465, *P* = .002) and NOMV (coef = 2.633, *P* = .011) were positively associated, whereas NOVC was negatively associated (coef = –1.141, *P* = .035). Other factors assessed in their respective models did not reach statistical significance (all *P* > .05).

## 4. Discussion

In this propensity score-matched study of patients with CKD5, we found that TOAU was independently associated with in-hospital mortality. This association was further supported by categorical analysis, with most categories showing statistically significant elevations in odds compared with no antibiotic use. Additionally, DOAU was also significantly associated with in-hospital mortality after multivariable adjustment, though the effect size was modest. PPA and CDOPA showed no statistically significant associations with mortality in the adjusted models.

To explore potential mechanisms underlying these associations, we examined factors independently associated with antibiotic use indicators. TOAU was significantly associated with TDF, NOPS, and NOVC. DOAU showed significant associations with TDF, ICU days, and NOPS.

### 4.1. Association between number of antibiotic types used and in-hospital mortality in patients with CKD5

In this propensity score-matched cohort of patients with CKD5, we observed a significant association between the TOAU and in-hospital mortality. This association persisted across multiple analytical approaches: when TOAU was analyzed as a continuous variable (Firth OR = 1.650, 95% CI: 1.148–2.492; *E*-value = 1.889), and when examined categorically, where most antibiotic type categories showed significantly elevated odds of mortality compared with no antibiotic use. The robustness of this finding was supported by bootstrap resampling and sensitivity analyses using standard logistic regression, which yielded consistent effect estimates. Additionally, DOAU was also significantly associated with in-hospital mortality after multivariable adjustment (OR = 1.081, 95% CI: 1.003–1.173), although the effect size was modest. This finding warrants validation in larger, adequately powered cohorts. Given the observational design of this study, causal relationships and underlying mechanisms cannot be definitively established. However, based on literature review and discussion among the research team, the potential mechanisms underlying this association may involve the following aspects:

1. Many antibiotics possess inherent nephrotoxic potential and are among the most common causes of drug-induced nephrotoxicity.^[13]^ Consistent with most published evidence, antibiotic use can induce renal injuries, including glomerular damage, tubular injury or dysfunction, and distal tubular obstruction, which further exacerbates disease progression in CKD5 patients.^[14,15]^ Additionally, glomerular filtration dysfunction leads to delayed antibiotic excretion, and the combination of multiple antibiotics may synergistically trigger nephrotoxic effects,^[13,16]^ ultimately contributing to in-hospital mortality.
2. Patients with CKD5 are inherently immunocompromised.^[17-19]^ Notably, the use of multiple antibiotic classes may further compromise the immune function of nephrology patients, which exacerbates already weakened immunity, increases susceptibility to infections, and ultimately initiates a vicious cycle of frequent antibiotic class switches.^[20,21]^ Previous studies have reported that CKD5 patients are characterized by recurrent hospitalizations, more frequent antibiotic use, and often polypharmacy, which is closely associated with immune system abnormalities in this patient population.^[22]^ Additionally, a study demonstrated that chronic kidney disease patients receiving long-term trimethoprim-sulfamethoxazole prophylaxis may develop variant immunodeficiency disorders.^[23]^ Collectively, antibiotic use imposes a burden on the intrinsic immunity of CKD5 patients, elevating the risk of infections and consequently contributing to increased mortality.
3. Furthermore, an increase in the number of antibiotic classes used in CKD5 patients is often accompanied by the empiric nature of therapy, which may mask the true infectious etiology and delay effective infection control.^[5,24,25]^ Through tracking the clinical diagnosis and treatment processes as well as antibiotic use data in nephrology departments, we found that the annual average rate of etiological testing before antibiotic administration was only approximately 38% to 52%. (see Table S6, Supplemental Digital Content 6, which illustrates the annual rates of pathogen testing prior to antimicrobial treatment in the Nephrology Department from 2022 to 2024.) The low etiological testing rate limits precise antibiotic selection, leading to a high proportion of empirical antibiotic use and frequent switches between antibiotic classes. Moreover, the combination of multiple antibiotics can induce the emergence of multidrug-resistant (MDR) bacteria, notably Clostridioides difficile, as well as other pathogens,^[22]^ which further deteriorates the patient’s condition and results in in-hospital mortality.

### 4.2. Our study did not find statistically significant associations between in-hospital mortality and PPA or CDOPA in patients with CKD5

A limited number of studies have failed to support an association between antibiotic dosage or duration and mortality in CKD5 patients. For instance, no correlation was observed between cephalosporin dosage and mortality risk in elderly CKD patients.^[26]^ However, the majority of studies indicate that CKD5 patients have impaired antibiotic metabolism due to the underlying disease, and the nephrotoxicity of antibiotics also restricts their use.^[7,27]^ Prescribing errors may induce nephrotoxicity, which can lead to in-hospital mortality in severe cases.^[28]^ To date, no studies have reported an association between PPA, CDOPA, and in-hospital mortality in CKD5 patients, and our results also do not support such associations. A potential explanation is the short duration of postoperative prophylactic antibiotic use, which may overlap with therapeutic antibiotic administration.

### 4.3. Factors associated with antibiotic use indicators

Analysis of factors influencing antibiotic use revealed that TOAU was positively associated with NOPS, TDF, and NOVC. In contrast, DOAU showed significant associations with TDF, ICU days, and NOPS.

The positive correlation between TOAU and NOPS emphasizes the critical importance of etiological testing prior to antibiotic administration. Identification of pathogenic bacteria enables targeted therapy with pathogen-sensitive antibiotics, which not only improves clinical treatment outcomes but also reduces the frequency of antibiotic switches, curbs the transmission of drug-resistant bacteria, and conserves healthcare resources. In China, the special campaign “Enhancing the Etiological Testing Rate Before Antibacterial Treatment in Hospitalized Patients” has been progressively advanced, with the testing rate integrated into the hospital quality assessment system. However, empirical antibiotic use remains prevalent in clinical practice. Consistent with this reality, our study demonstrated positive correlations between TOAU and TDF, as well as between TOAU and NOVC. The association with TDF likely reflects the common clinical practice of initiating or switching antibiotics in response to persistent fever, while the association with NOVC may indicate that patients requiring venous catheterization are more susceptible to healthcare-associated infections, prompting broader empirical coverage. Follow-up interviews with clinicians confirmed that antibiotic initiation is often determined based on nonspecific clinical manifestations, such as fever (reflected by TDF). Notably, neither TDF nor NOVC serves as a specific indicator for bacterial infection. Empirical antibiotic selection based on such nonpathogenic indicators may lead to frequent antibiotic class switches and an increased number of antibiotic classes administered.

Regarding DOAU, its associations with TDF, ICU days, and NOPS suggest that prolonged antibiotic therapy is primarily driven by infection severity and the need for intensive care. Patients with longer fever duration, greater pathogenic burden, and ICU admission inherently require extended treatment courses, which may explain the observed association between DOAU and mortality in our study, albeit with a modest effect size.

Furthermore, clinicians frequently rely on fever status to decide on antibiotic discontinuation. In conclusion, nonspecific clinical indicators including TDF and NOVC, along with pathogenic burden reflected by NOPS, were associated with an increase in the number of antibiotic classes used. Our findings indicate that empirical antibiotic use based on nonspecific indicators such as TDF and NOVC, in the absence of confirmed pathogens, is common in the clinical management of patients with CKD5. These observations suggest that improving the etiological testing rate before antibiotic administration and limiting the number of antibiotic types used may represent reasonable elements of more cautious antibiotic stewardship. However, these findings warrant validation in larger prospective cohorts.

## 5. Conclusions

In this propensity score-matched study of patients with CKD5, TOAU was significantly associated with in-hospital mortality. This association persisted across multiple analytical approaches, including continuous and categorical analyses, and demonstrated robustness to unmeasured confounding (*E*-value = 1.889). Additionally, DOAU was also significantly associated with mortality, although the effect size was modest. In contrast, no statistically significant associations were observed between in-hospital mortality and PPA or CDOPA. Analysis of factors influencing antibiotic use revealed that TOAU was positively associated with TDF, NOPS, and NOVC, while DOAU was positively associated with TDF, ICU days, and NOPS.

These results highlight the potential importance of more cautious antibiotic stewardship in patients with CKD5, including improving etiological testing before antibiotic administration and limiting unnecessary increases in the number of antibiotic types. Given the observational design and relatively small number of outcome events in this study, these findings warrant validation in larger, adequately powered prospective cohorts.

## 6. Strengths and limitations

### 6.1. Strengths

This study has several strengths. First, to mitigate confounding bias, we employed PSM to balance key baseline characteristics – including age, gender, admission year, and number of admission surgeries – between the in-hospital death group and the survival group, thereby reducing selection bias and enhancing internal validity. Second, given the small number of outcome events (31 deaths after matching), we applied Firth penalized logistic regression to reduce small-sample bias and address separation issues, and we performed bootstrap resampling to provide nonparametric uncertainty estimates. Third, we conducted sensitivity analyses using standard logistic regression in the PSM‑matched cohort and calculated *E*-values to assess robustness to unmeasured confounding; the *E*-value for TOAU (1.889) suggested moderate robustness against potential unmeasured confounders. To the best of our knowledge, this is the first study to identify a significant association between TOAU and in-hospital mortality in patients with CKD5.

### 6.2. Limitations

Several limitations should be acknowledged. First, as a single-center retrospective study, the sample representativeness is constrained by regional characteristics and local clinical practice patterns, which may limit the generalizability of our findings to other healthcare settings. Second, the observational design precludes causal inference; while we identified significant associations between antibiotic use indicators and mortality, reverse causation and residual confounding cannot be fully excluded. Third, the small number of outcome events after matching (31 deaths) limited our statistical power, particularly for detecting modest effect sizes and for examining interactions or subgroup effects. The wide CIs for some estimates, especially in the categorical TOAU analysis, reflect this uncertainty. Fourth, the timing of antibiotic initiation relative to infection onset and the temporal relationship between antibiotic switches and clinical deterioration could not be established, introducing potential time-related biases. Finally, our antibiotic use indicators (TOAU, DOAU, PPA, CDOPA) may not capture the full complexity of antibiotic prescribing, including the choice of specific agents, dosing appropriateness, and de-escalation practices. It is important to note that our hospital has an established clinical pharmacy department. In the nephrology unit, all antibiotic prescriptions require consultation with a clinical pharmacist, who provides a regimen tailored to the patient’s renal function and clinical condition. However, this institutional context does not confirm that dosing was appropriate in individual cases. Therefore, renal-dose appropriateness was not directly assessed in this study, and this remains a limitation.

## 7. Future research directions

Future studies should adopt multicenter prospective cohort designs to dynamically collect data, enabling analysis of the temporal relationship between antibiotic use and mortality outcomes. Additionally, integrating laboratory‑based mechanistic studies is essential to validate causal links, ultimately providing stronger evidence‑based support for the management of in‑hospital mortality in CKD5 patients.

## Acknowledgments

We thank all participants in the study for their invaluable contributions.

## Author contributions

**Conceptualization:** Qiantu Hu, Xuejiao Fan, Qianni Huang, Limei Ou.

**Data curation:** Qiantu Hu, Qiuhua Huang, Yongxue Huang, Suzhen Wei, Qianni Huang.

**Formal analysis:** Qiantu Hu, Feixiang Lao, Qianqian Luo.

**Funding acquisition:** Limei Ou.

**Investigation:** Qiantu Hu, Qiuhua Huang, Feixiang Lao, Yongxue Huang, Zhongqiu Mei.

**Methodology:** Qiantu Hu, Xuejiao Fan, Qianqian Luo.

**Supervision:** Xuejiao Fan, Limei Ou.

**Validation:** Qiantu Hu.

**Visualization:** Qiantu Hu.

**Writing – original draft:** Qiantu Hu.

**Writing – review & editing:** Suzhen Wei, Limei Ou.












